# Doped Nanoscale NMC333 as Cathode Materials for Li-Ion Batteries

**DOI:** 10.3390/ma12182899

**Published:** 2019-09-07

**Authors:** Ahmed M. Hashem, Ashraf E. Abdel-Ghany, Marco Scheuermann, Sylvio Indris, Helmut Ehrenberg, Alain Mauger, Christian M. Julien

**Affiliations:** 1 National Research Centre, Inorganic Chemistry Department, 33 El Bohouth St., (former El Tahrir St.), Dokki-Giza 12622, Egypt; ahmedh242@yahoo.com (A.M.H.); achraf_28@yahoo.com (A.E.A.-G.); 2 Karlsruhe Institute of Technology (KIT), Institute for Applied Materials (IAM), P.O. Box 3640, 76021 Karlsruhe, Germany; macolino@gmx.de (M.S.); sylvio.indris@kit.edu (S.I.); helmut.ehrenberg@kit.edu (H.E.); 3 Institut de Minéralogie, Physique des Matériaux et de Cosmochimie (IMPMC), Sorbonne Université, Campus Pierre et Marie Curie, UMR 7590, 4 place Jussieu, 75252 Paris, France; alain.mauger@upmc.fr

**Keywords:** sol-gel synthesis, EDTA chelator, cathode materials, layered oxide, doping, lithium-ion batteries

## Abstract

A series of Li(Ni_1/3_Mn_1/3_Co_1/3_)_1−x_*M*_x_O_2_ (*M* = Al, Mg, Zn, and Fe, *x* = 0.06) was prepared via sol-gel method assisted by ethylene diamine tetra acetic acid as a chelating agent. A typical hexagonal α-NaFeO_2_ structure (*R*-3*m* space group) was observed for parent and doped samples as revealed by X-ray diffraction patterns. For all samples, hexagonally shaped nanoparticles were observed by scanning electron microscopy and transmission electron microscopy. The local structure was characterized by infrared, Raman, and Mössbauer spectroscopy and ^7^Li nuclear magnetic resonance (Li-NMR). Cyclic voltammetry and galvanostatic charge-discharge tests showed that Mg and Al doping improved the electrochemical performance of LiNi_1/3_Mn_1/3_Co_1/3_O_2_ in terms of specific capacities and cyclability. In addition, while Al doping increases the initial capacity, Mg doping is the best choice as it improves cyclability for reasons discussed in this work.

## 1. Introduction

Lithium-ion batteries (LIBs) applied to power different systems such as portable electronics and electric cars require high-power density, fast charge/discharge rates, and long cycling lives [1,2,3]. It is well known that the positive electrode (cathode) in LIBs plays an essential role in guiding the electrochemical performance and is considered as the limiting element of the battery. Used in the first generation of LIBs, the layered oxide LiCoO_2_ (LCO) crystallizing with the α-NaFeO_2_ type structure [4] demonstrated good reversibility and high-rate capability making this material very popular in commercial lithium-ion batteries. However, some drawbacks have been identified including high cost, toxicity, limited practical capacity (~130 mAh g^−1^; Δ*x*(Li) ≈ 0.5 in the voltage range 2.7–4.2 V), poor thermal stability, and fatigue on crystal structure, which will limit further use of LCO in the forthcoming years [5,6,7].

Intensive studies for the replacement of LCO were conducted by formulating layered oxides with multi-components of the system Li*M*O_2_ (*M* = Ni, Mn, and Co); then, the new lamellar compounds LiNi_1-y_Mn_y_O_2_ (NMO), LiNi_1−y_Co_y_O_2_ (NCO), and LiNi_y_Mn_z_Co_1__−y__−z_O_2_ (NMC) were successively proposed [8,9,10,11,12,13]. The performance of LiNi_x_Mn_y_Co_1__−x__−y_O_2_ as cathode material relies on the distribution of the transition-metal (TM) cations. For *y* = *z*, the Ni and Mn ions are, respectively, in 2+ and 4+ oxidation state. Ni^2+^ ions (r_(Ni2+)_ = 0.69 Å) are active species, while Mn^4+^ ions (*r*_(Mn4+)_ = 0.54 Å) stabilize the structure of the α-NaFeO_2_ phase [14]. However, high Mn content in the NMC composition may cause phase transition from the layered to the spinel structure [15]. Co^3+^ ions (*r*_(Co3+)_ = 0.545 Å) play a role in reducing the cation mixing, corresponding to the anti-site defect where Ni^2+^ and Li^+^ ions exchange their site (interlayer mixing). This defect results from the fact that the ionic radius of Li^+^ (0.74 Å) is close to that of Ni^2+^ [16,17]. Therefore, the content of transition-metal cations in LiNi_x_Mn_y_Co_1__−x__−y_O_2_ should be optimized to give the best performance as an active cathode material in lithium batteries. In this framework, the composition LiNi_1/3_Mn_1/3_Co_1/3_O_2_ (named NMC333 hereafter) is of particular interest, as it delivers a reversible capacity of approximately 200 mAh g^−1^ in the voltage range 2.8–4.6 V versus Li^+^/Li [18,19]. Despite the advantage of high capacity, these materials display the shortcoming of capacity fading when cycled at high voltage and high-rate current density [19]. Two effective approaches have been successfully used to improve their electrochemical performance: Addition of substituting element (doping) and surface modification (coating) [20,21]. Fergus [22] reviewed the effect of doping on the electrochemical performance of cathode materials pointing out that the analysis of doping effects is complicated by the dopant–host structure interrelations modifying the microstructure and morphology. Appropriate metal doping will improve the structural integrity of the oxide framework and hinders oxygen release, because of the higher *M*–O bond dissociation energy in the *M*O_6_ octahedron [23].

Many attempts have been made to insert TM ions in NMC frameworks using various elements such as other TM ions (Ti, Zr, Nb, Fe, Cr, Cu) [24,25,26,27,28,29,30], rare earths (La, Ce, Pr, Y) [31,32,33,34], post-transition metal (Zn) [35], and *sp* elements (Al, Mg), which are the most popular dopants [36,37,38,39]. Depending on the nature of the substituting atom, the crystal structure and electrochemical performance of the electrode are differently modified. Substitution of Al and Mg for TMs in NMCs enhances the thermal stability and improves the electrochemical stability of this cathode material [40,41,42]. Mg doping has improved NMC333 electrodes by modifying the microstructure and reducing the charge transfer resistance [29]. Studies of the impact of Co-selective substitution by Ti, Al, and Fe showed that ~8% Ti^4+^ improves the rate capability, ~5% Al^3+^ improves the capacity retention, while Fe^3+^ doping is detrimental to the electrochemical performance due to the increase of concentration of anti-site defects (implying a *c/a* ratio reduction) resulting in kinetic limitations in NMC333 [28,36]. Aluminum is a very commonly used substituting element in NMC333 cathode materials [28,29,43,44,45]. Contrary to the expectation of a decreased capacity as Al^3+^ ions are not active in the considered electrochemical window (cannot show further oxidation), one observes better performance due to the improved electrode kinetics, structural modifications, and microstructural effects. However, it must be pointed out that the major differences compared to other reported studies come from the different preparation methods producing particles with various morphologies in connection with the shape of grains, particle size, specific surface area, and particle size distribution. Numerous works have shown that wet-chemistry is a powerful technique to optimize morphology of oxides; thus, using ethylene diamine tetra acetic acid (EDTA) as the chelating agent during the sol-gel synthesis may be unique in this study.

In this work, we investigated the effect of a partial substitution of the TMs on structural, morphological, and electrochemical properties of a series of Li(Ni_1/3_Mn_1/3_Co_1/3_)_0.94_*M*_0.06_O_2_ cathode materials with divalent Zn^2+^, Mg^2+^, and trivalent Fe^3+^ and Al^3+^ ions. The samples were synthesized using a sol-gel method assisted by EDTA as chelator. Ethylene diamine tetra acetic acid, which can chelate several metal ions at the same time, has a unique property of reducing the calcination temperature for the oxide preparation. Recently, we reported the efficiency of this method to prepare nanostructured LiMn_2_O_4_ powders with well-controlled particle size and size distribution [46]. The reason is that EDTA acts as a hexadentate ligand including six sites (i.e., two amines and four carboxyl groups) that can bind to the metal ions. The EDTA forms stable and strong complexes with metal ions through strong masking of free metal ions, which alleviates negative effects such as precipitation of sparingly soluble salts. The strong chelating power of EDTA makes possible the accurate control of dopant concentrations with accuracy better than 1%. Attention has been paid to synthesize all the samples with almost the same morphology and particle size, so that direct comparison of the electrochemical properties of NMC333 cathodes doped with Al, Mg, Fe and Zn is meaningful.

## 2. Materials and Methods

Li(Ni_1/3_Co_1/3_Mn_1/3_)_1−x_*M*_x_O_2_ (*M* = Fe, Al, Mg and Zn, *x* = 0.06) materials were prepared by a sol-gel method EDTA as a chelating agent. Li, Ni, Mn, Co, Mg, Zn, and Al acetates and iron citrate (Merck KGaA, Darmstadt, Germany, 99.99% grade) were used as starting materials. Proper amounts of these starting materials, according to the desired stoichiometry, were dissolved in de-ionized water to form aqueous solution. The dissolved solutions were added stepwise into a stirred aqueous solution of EDTA with a 1:1 metal/chelator ratio. The solution was stirred for 3 h for a homogenous mixture of the reaction reagents and favor complex reaction between metal ions and EDTA. Ammonium hydroxide was added to adjust pH of the solution at ~7. Transparent gel was formed after slow evaporation of the solution. The resulting precursor was heated and decomposed at 450 °C for 5 h in air then ground and recalcined at 700 °C for 8 h in air.

The crystal structure of the prepared materials was investigated by X-ray diffraction (XRD) using Philips X’Pert apparatus (Hamburg, Germany) equipped with CuKα X-ray source (λ = 1.5406 Å). X-ray diffraction measurements were collected in the 2θ range 10–80° at low scanning rate. Sample morphology was observed by scanning electron microscopy (SEM; JEOL model JEM-1230, Freising, Germany). Fourier transform infrared (FTIR) spectra were recorded with a vacuum interferometer (model IFS 113 (Bruker, Karlsruhe, Germany). In the far-infrared region (800–100 cm^−1^), the vacuum bench apparatus was equipped with a 3.5 μm-thick Mylar beam splitter, a globar source, and a DTGS/PE far-infrared detector. Raman scattering (RS) spectra were measured using a micro-Raman-laser spectrometer model Lab-Ram (Horiba-Jobin-Yvon, Longjumeau, France) equipped with 50× microscope lens, D2 filter, aperture of 400 μm, and a slit of 150 μm. The spectra have been recorded with the red (λ = 632 nm) laser excitation. ^57^Fe Mössbauer spectroscopic measurements were performed in transmission mode at room temperature using a constant acceleration spectrometer with a ^57^Co (Rh) source. Isomer shifts are given relative to that of α-Fe at room temperature. ^7^Li magic-angle spinning (MAS) NMR was performed on a Bruker Avance 200 MHz spectrometer (*B*_0_ = 4.7 T) using 1.3 mm zirconia rotors in a dry nitrogen atmosphere. An aqueous 1 mol L^−1^ LiCl solution was used as the reference for the chemical shift of ^7^Li (0 ppm). ^7^Li one-dimensional magic angle spinning nuclear magnetic resonance (MAS NMR) experiments were performed at 298 K and a spinning speed of approximately 60 kHz with a rotor synchronized Hahn-echo sequence (π/2–τ–π–τ– acquisition). The typical values for the recycle delay and the π/2 pulse length were 1 s and 2 μs, respectively.

Electrochemical tests were performed using a multichannel potentiostatic-galvanostatic system VMP3 (Biologic, Grenoble, France). The cathode mixture for the fabrication of the positive electrode was prepared by mixing 80 wt.% of the active material with 10 wt.% of super P^®^ Li carbon (TIMCAL) and 10 wt.% of polyvinylidene fluoride binder (PVDF), dissolved in N-methylpyrrolidone (NMP). The electrode was formed by loading this mixture at 6 mg cm^−2^ onto an Al foil dried at 100 °C for 1 h in a vacuum oven. Coin cells were assembled in an argon-filled dry box with Li foil as anode and glass-fiber separator soaked with 1 mol L^−1^ LiPF_6_ in ethylene carbonate/dimethyl carbonate (EC/DMC) (1:1 by v/v) aprotic solution. Galvanostatic charge-discharge cycling was carried out at C/10 rate in the voltage range 2.5–4.5 V versus Li^+^/Li^0^.

## 3. Results and Discussion

### 3.1. Structural Analysis

**Figure 1:**
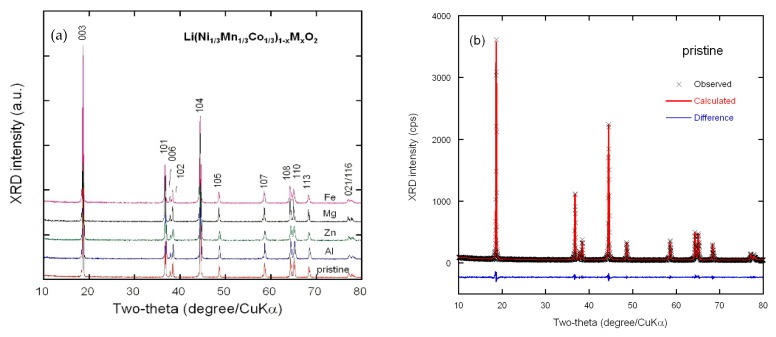

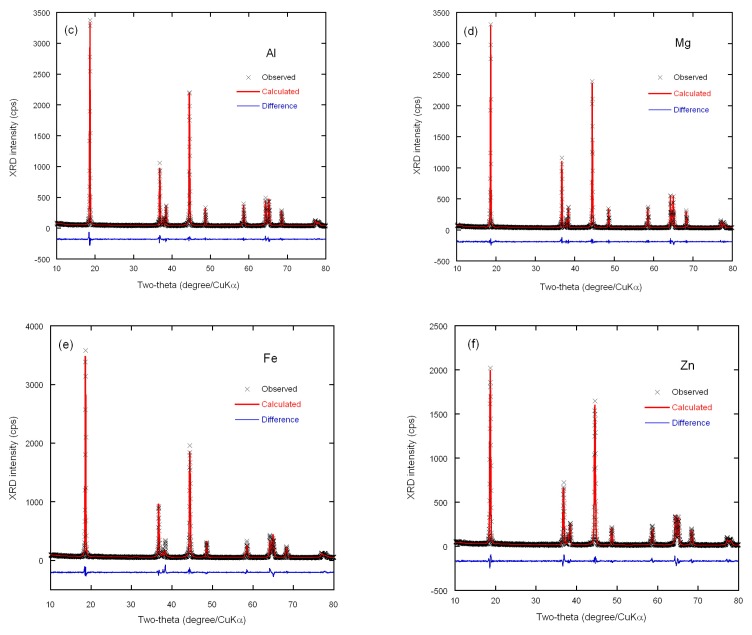
X-ray diffraction (XRD) patterns (**a**) and Rietveld refinements (**b**–**f**) of undoped Li(Ni_1/3_Mn_1/3_Co_1/3_)O_2_ and doped Li(Ni_1/3_Mn_1/3_Co_1/3_)_1−x_*M*_x_O_2_ (*x* = 0.06, *M* = Fe, Al, Mg, Zn), respectively. Cross marks are experimental data and solid lines (in red) are calculated diagrams. The curve at the bottom (in blue) is the difference between the calculated and observed intensities.

The XRD diagrams of pristine LiNi_1/3_Mn_1/3_Co_1/3_O_2_ and doped Li(Ni_1/3_Mn_1/3_Co_1/3_)_1−x_*M*_x_O_2_ powders (*M* = Fe, Al, Mg, Zn; *x* = 0.06) are presented in Figure 1a. We can clearly observe well-separated XRD reflections for all prepared samples with very smooth background, indicating highly crystallized products. These diffraction peaks are indexed to a hexagonal lattice α-NaFeO_2_-type (*R*-3*m* space group, standard card JCPDS 82-1495) without any impurity phase, reflecting the formation of a single-phase layered structure. This unique and well-developed layered structure can be confirmed from the distinct splitting of the 108/110 and 006/102 doublets. Values of the lattice parameter *a* (which related to the average metal-metal intraslab distance) and *c* (which related to the average interslab distance) calculated for parent and doped NMC333 are summarized in Table 1. The lattice parameters of parent LiNi_1/3_Mn_1/3_Co_1/3_O_2_ were consistent with previous reported results [36,47]. The material being an ionic compound, the dopant ions were inserted preferentially on sites that minimize the cost in Coulomb energy. As a first consequence, none of the dopants were expected to substitute for Mn^4+^. Mg and Zn being divalent were substituted for Ni^2+^. As we shall see later in this work, the Mössbauer results showed that iron was introduced in the matrix in the low-spin Fe^3+^ state. Therefore, according to the rule mentioned above, both Al^3+^ and Fe^3+^ substitute for Co^3+^. The Rietveld refinements based on the XRD patterns were done accordingly, including the possibility for the dopants to create also an anti-site defect. Indeed, an important character of these layered rock–salt structures is the cation mixing, occupation of the 3*b* Li interlayer sites by Ni^2+^ ions in the pristine NMC333, and possibly also by dopant metal ions. The results of the refinement are displayed in Figure 1b–f and data listed in Table 1. The residual and agreement parameters (*R* and χ^2^) of the Rietveld refinement were very good using this model, taking into account such a cation mixing. 

By introducing Mg^2+^ ions in the structure, the lattice expansion occurred in both the *a* and *c* directions, due to the slightly larger radius of Mg^2+^ (*r*_(Mg2+)_ = 0.72 Å) compared to that of Ni^2+^ (*r*_(Ni2+)_ = 0.69 Å). Reciprocally, the ionic radius of Al^3+^ (*r*_(Al3+)_ = 0.53 Å) was close to but a little bit smaller than that of low-spin Co^3+^ (*r*_(Co3+)_ = 0.545 Å), so that the substitution mainly induces a small shrinking of the lattice parameters. In both cases, however, the lattice distortion is small since the ionic radii are close. On the contrary, the radius of Fe^3+^ (*r*_(Fe3+)_ = 0.645 Å) was much larger than that of Co^3+^, even larger than high-spin Co^3+^. Nevertheless, there is a general agreement for the introduction of Fe^3+^ (*r*_(Fe3+)_ = 0.645 Å) on Co^3+^ positions [23,28,36,48], so that the rule mentioned above remains valid, despite the strong lattice distortion that this substitution will cost. The substitution of Fe^3+^ for Co^3+^ leads a lattice expansion, i.e., 0.4% and 0.2% for the *a*- and *c*-axis, respectively. On another hand, the *c*/*a* ratio was reduced, which suggests an increase in the anti-site cation defect concentration [28]. In the same way, a large lattice distortion was expected with the substitution of Zn^2+^ for Ni^2+^, since the size of Zn^2+^ (*r*_(Zn2+)_ = 0.74 Å) was bigger than that of Ni^2+^. One then would expect an expansion of the unit-cell parameters as in the case of Mg^2+^ and Fe^3+^. However, for Zn^2+^ doping, the data in Table 1 show that the *a*-parameter slightly increased, while the *c*-lattice parameter was not affected. This abnormal behavior provides evidence of important lattice distortions associated to the incorporation of Zn^2+^. It is also the indication of the degree of cation mixing in 3*a* and/or 3*b* sites, in agreement with Table 1. 

It should be pointed out that the increase in the degree of cation mixing is known to limit kinetics that cause poor rate capability of the Fe-doped electrode. However, the ionic radius (*r*_(Mg2+)_ = 0.72 Å) of Mg^2+^ being close to that of Li^+^ (*r*_(Li+)_ = 0.76 Å), the anti-site defect corresponding to a local exchange Mg–Li is likely, i.e., a small fraction of the Mg^2+^ ions will occupy the Li-sites. 

The cation mixing can be detected by different ratios. First, the *c/a* lattice ratio indicates the deviation of the rock–salt structure (i.e., *c/a* > 4.90). Higher value of peak ratio *I*_003_/*I*_104_ is the second indicator for a lower amount of undesirable cation mixing and better hexagonal structure [49]. The *R* factor (*R* = (I_006_ + I_102_)/I_101_) is the third fingerprint of the hexagonal ordering; the lower the *R*, the better the hexagonal ordering [50,51]. However, note that rules concerning *I*_003_/*I*_104_ and *R* are well established for undoped NMC samples. The distortion of the lattices associated to the doping may also affect these parameters. For instance, we see in Table 1 that, according to the more reliable Rietveld refinement results, samples doped with Mg and Al have lower cation mixing with well-ordered rhombohedral structures, despite the fact that *I*_003_/*I*_104_ in the Al-doped sample was smaller than in the pristine sample.

The doping by any of the elements investigated reduces the concentration of Ni^2+^–Li anti-site defects. In particular, the introduction of Mg ions on 3*a* sites led to a remarkable decrease of Ni^2+^ ions on the 3*b* site (Table 1); this observation matches well with previous reports [52,53]. However, while most of the Mg, Fe, and Zn ions were the 3*a* sites, the Rietveld refinement showed that the 3*b* Li site was also partly occupied by these foreign ions. This feature is attributable to the fact that the ionic radii of Mg^2+^, Fe^3+^, and Zn^2+^ are comparable to that of Li^+^ (*r*_(Li+)_ = 0.76 Å) [54]. On the one hand, the much smaller ionic radius of Al^3+^ can explain the absence of Al^3+^ ions on the lithium site. Overall, the cation mixing obtained by adding the concentration of nickel and dopant ions on the 3*b* lithium sites is 1.4% and 1.5% for Al- and Mg-doped samples, respectively, smaller than 2.4% in the undoped sample. On the other hand, this concentration was larger in the Zn- and Fe-doped samples. 

Two other structural parameters can be deduced from Rietveld refinements: *I(LiO_2_)* the thickness of the inter-slab space and *S(MO_2_)* the thickness of the metal–O_2_ planes [55]. As seen from Table 1, both *a*- and *c*-parameters and *S*(*M*O_2_) were minimum and *I*(LiO_2_) was maximum for *sp*-doped elements, which confirms that these samples had better structural integrity.

The cation mixing between metal ions (Ni^2+^ and *M*^2+ or 3+^) and Li ions on the two sites resulted in two competitive effects on lattice parameter depending on the ionic radius and ionic charge of metal: (i) As *r*(Li^+^) was larger than the ionic radii of the other Ni, Mn, and Co ions, this difference favored an increase of the in-plane parameter *a* due to the presence of Li ions on the 3*a* site. Moreover, the Li^+^ ions carried only a charge +1, so that the occupation of 3*a* sites by Li^+^ decreased the repulsive Coulomb potential with the neighboring TM ions inside the slabs. This effect also favored an increase of the *a*-lattice parameter, (ii) this effect is in part compensated by the concomitant presence of the Ni^2+^ and M^2+ or 3+^ on the 3*b* site. Moreover, the metal ions on the 3*b* site carried more charge than Li^+^, which leads to a stronger electrostatic attraction between these ions and O^2–^ in the interslab plane. As a consequence, the Li-O interslab distance and the related *c*-lattice parameter decreased. This stronger Li–O bond also hinders Li^+^ diffusion through the NMC framework, so that the Li^+^ ions on the 3*a* sites do not contribute to the electrochemical process [17].

**Figure 2:**
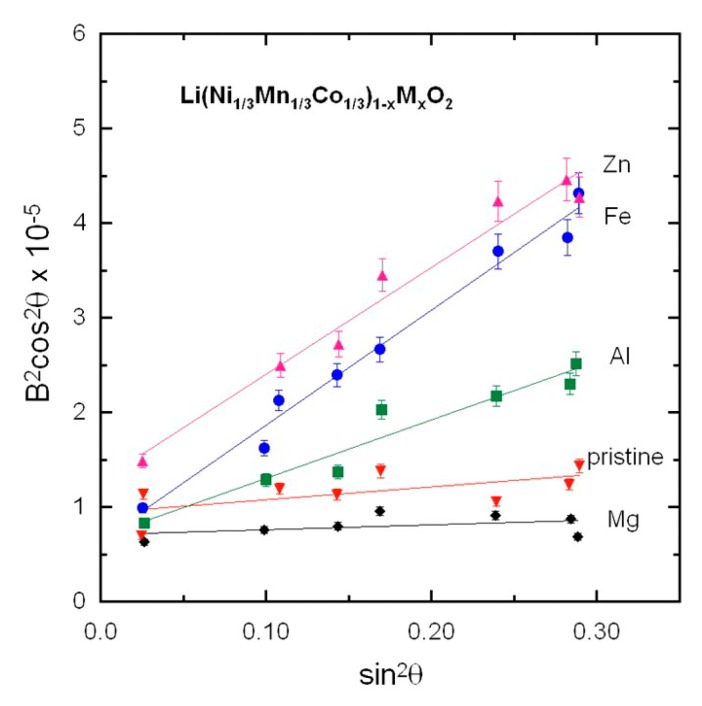
Analysis of the full-width at half-maximum, *B*, of XRD peaks according to Equation (1). *B* is expressed in radian.

The broadening of reflections is an indicator not only to the crystallinity of the Li(Ni_1/3_Mn_1/3_Co_1/3_)_1−x_M_x_O_2_ powder but also to the local deformation of the structure. The combination of the Scherrer’s equation for crystallite size with the Bragg’s law for diffraction leads to Equation (1):

(1) $$B^{2}\cos^{2}\theta =6\langle e^{2}\rangle \sin^{2}\theta +\frac{K^{2}\lambda^{2}}{L_{c}}$$

which can be used to determine coherence length *L*_c_ and micro-strain field <*e*^2^>. *B* is the full-width at half-maximum (FWHM) in radian, θ is the diffraction angle, and *K* is a near-unity constant related to crystallite shape. The first member is reported as a function of sin^2^θ in Figure 2 for the pristine and doped Li(Ni_1/3_Mn_1/3_Co_1/3_)O_2_ samples. The plots are well fit by straight lines, in agreement with Equation (1). The slope of the lines gives the value of the strain field <*e*^2^>, while the coherence length *L*_c_ is given by the extrapolation to sin θ = 0. For all investigated samples, values were in the range 15 ≤ *L*_c_ ≤ 25 nm. On the other hand, we found that <*e*^2^> was strongly affected by the nature of the doping elements. While the strain field was negligible in the undoped and Mg-doped samples, it rose to <*e*^2^> = 0.4221 × 10^−5^ rd^2^ in Al-doped samples, and became as large as 0.8743 × 10^−5^ and 0.8458 × 10^−5^ rd^2^ in Fe- and Zn-doped samples, respectively. 

The negligible value of <*e*^2^> in Mg-doped samples proves that Mg stabilizes the structure of the lattice, a result that is consistent with the fact that it almost totally eliminated the Li-Ni anti-sites. <*e*^2^> is non-negligible in Al-doped samples. This is also consistent with the fact that the concentration of Li-Ni anti-sites, although smaller than in the pristine sample, was not eliminated by the Al doping. This result gives evidence of a better structural stability of the Mg-doped sample than the Al-doped sample. For the Zn- and Fe-doped cases, the large values of <*e*^2^> imply important lattice distortions that will further weaken the structural stability of Fe- and Zn-doped samples, as expected from the discussion reported above on the lattice parameters and the consideration of ionic radii. 

### 3.2. Morphology

**Figure 3:**
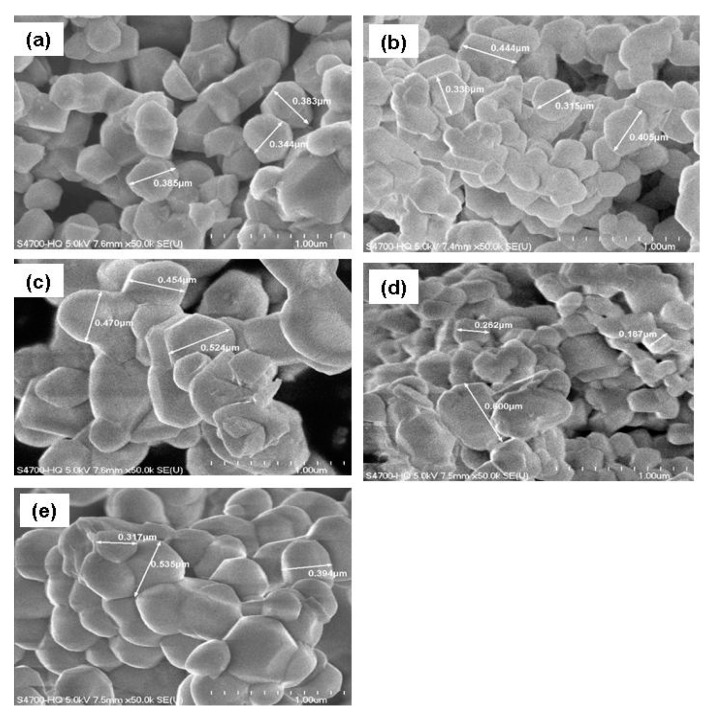
Field-emission SEM images of (**a**) undoped and (**b**–**e**) *M*-doped NMC333 powders with *M* = Al, Mg, Fe, Zn, respectively.

Figure 3 presents the SEM images of the undoped- and doped-NMC333 materials. The nanoscale powders consisted of hexagonally like shaped particles with flat facets. As the morphology is one of the main factors which affects the electrochemical performance of the electrode materials, we tried to maintain identical aspects for all the samples. However, although the synthesis conditions were the same, the particle size and morphologies depended on the doping element because of the change in the reaction equilibrium despite similar temperature, pH value, etc. The undoped NMC333 showed regular particles, 300–500 nm in size, with quite narrow size distribution (Figure 3a). The Al-doped sample became more uniform and exhibited smaller primary particles with size lying between 200 and 450 nm (Figure 3b). The Mg-substituted sample showed larger particle sizes (Figure 3c). The Fe-doped sample had a broad size distribution with particles in the range 100–600 nm (Figure 3d). The Zn-doped material presented less faceted particles with a narrower size distribution (i.e., 300–500 nm); particles tended to form microspheres (Figure 3e). 

All powders maintain the initial morphology of the undoped sample, except a tendency of agglomeration in the case of Fe-doped sample (Figure 3e). It seems that the formation of agglomerates depends strongly of the synthesis technique. Ren et al. [38] reported agglomeration of Al-doped NMC powders even at low concentration of aluminum (*y* = 1/12) when prepared by solvent evaporation method. On another hand, Lin et al. [43] showed well-dispersed particles with a slight decrease of grain size for moderated Al-doped powders (*y* = 0.1) obtained via sol-gel method using polyvinyl alcohol as organic fuel. In conclusion, all these NMC oxides showed almost well-dispersed primary particles with relatively bright and clear surface; in addition, the particles did not display a change in surface roughness with doping. The absence of aggregated particles was attributed to the synthesis process via EDTA chelating assistance with an efficient pH control.

**Figure 4:**
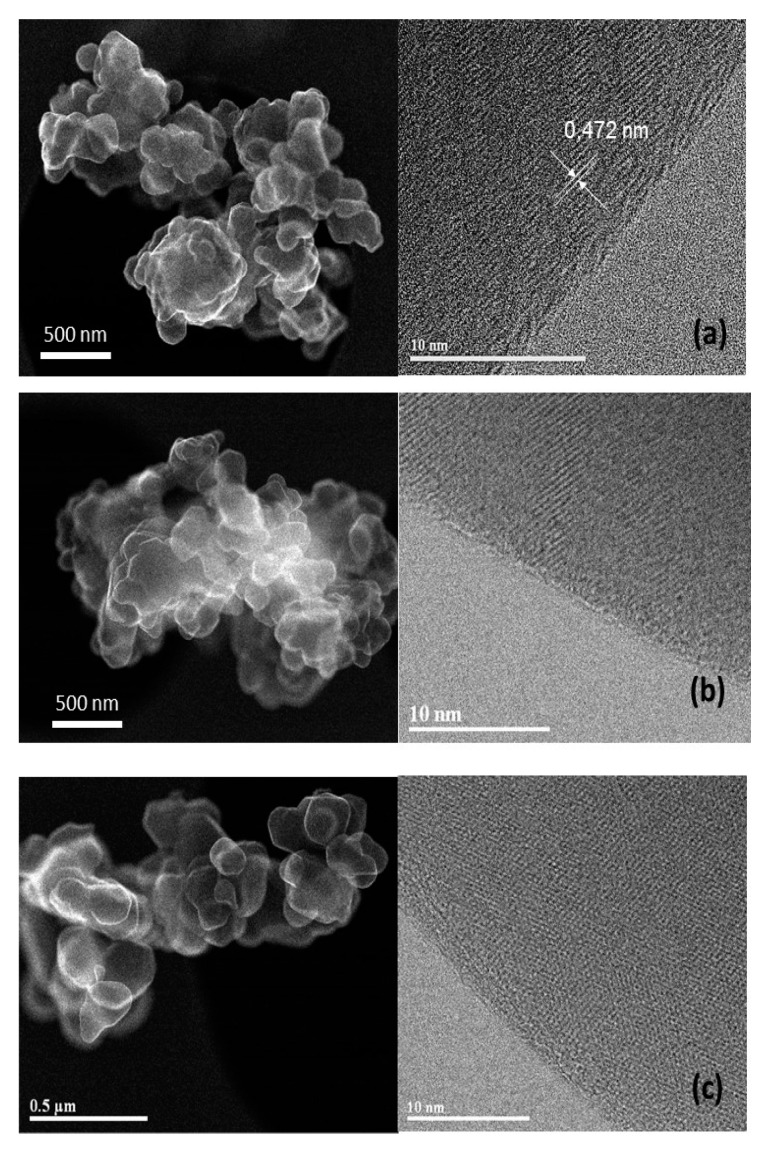
TEM images of (**a**) undoped, (**b**) Al-doped, and (**c**) Mg-doped NMC333 samples. The HRTEM micrographs reveal well-defined lattice fringes with a separation of 4.72 Å corresponding to the (003) plane.

To further examine the sample morphology, Figure 4 presents the typical TEM images of undoped (a) and Al- (b) and Mg-doped NMC333 samples (c) confirming the submicronic size of the particles. The high magnification TEM (HRTEM) micrographs of individual nanoparticle reveal well-defined lattice fringes with a separation of 4.72 Å corresponding to the (003) plane. One also observes that the edges of all as-crystallized particles were well defined (i.e., without disordered surface layer). Therefore, as we shall see later, the difference of morphology among the different samples was small enough to allow for a direct comparison of their electrochemical properties.

### 3.3. Local Structure

**Figure 5:**
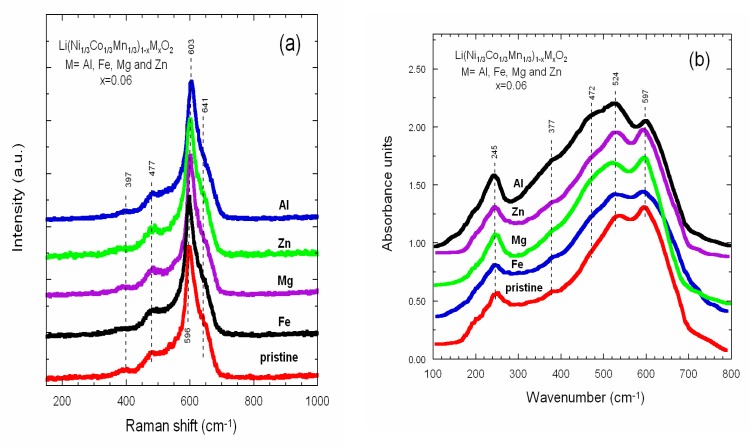
(**a**) Raman spectra and (**b**) FTIR of undoped and doped LiNi_1/3_Mn_1/3_Co_1/3_O_2_ (*x* = 0.06) powders synthesized via sol-gel method.

The local structure, i.e., short-range environment of lithium within NMC materials was investigated using several analytical methods: Raman, FTIR and Mössbauer spectroscopies, and ^7^Li NMR measurements. Figure 5a,b display the Raman and FTIR spectra of NMC333 samples, respectively. Considering the layered *R*-3*m* structure (D^5^_3d_ spectroscopic symmetry) for LiNi_1/3_Mn_1/3_Co_1/3_O_2_, one expects six Raman active modes (3*A*_1g_ + 3*E*_g_) and seven IR active modes (4*A*_2u_ + 3*E*_u_) [56]. As a general trend, the doping did not bring significant alteration in the band positions in both vibrational spectra. Characteristic Raman bands were recorded at 397 and 477 cm^−1^ (O–M–O bending vibrations) and 603 and 641 cm^−1^ (M–O symmetrical stretching), while FTIR patterns were measured at 245 cm^−1^ (Li cage mode), 377 and 472 cm^−1^ (O–M–O asymmetric bending modes), and 527 and 594 cm^−1^ (asymmetric stretching modes of *M*O_6_ octahedra). In conclusion, doping did not provoke significant change in spectral features, except a slight broadening of the Li cage mode, which reflects the degree of cationic mixing in the interlayer space. 

**Figure 6:**
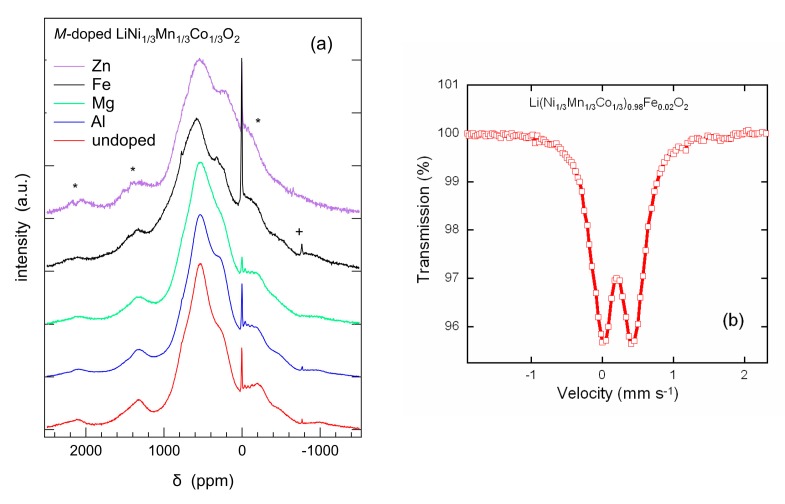
(**a**) ^7^Li MAS NMR spectra of undoped and doped LiNi_1/3_Mn_1/3_Co_1/3_O_2_ samples; (**b**) Fe Mössbauer spectrum of Li(Ni_1/3_Mn_1/3_Co_1/3_)_1−x_Fe_x_O_2_.

Solid-state ^7^Li-MAS NMR measurements were carried out to study the structural properties on a local scale, knowing that paramagnetic ions in the surrounding of the lithium ions have strong effects on the NMR spectra due to the Fermi-contact mechanism, i.e., the transfer of spin density from the unpaired electrons of the paramagnetic ions to the lithium nucleus. In LiNi_1/3_Mn_1/3_Co_1/3_O_2_, Co^3+^ is in its low-spin state and not magnetic, but both Ni^2+^ and Mn^4+^ contribute to the resulting overall hyperfine shifts. Figure 6a shows the ^7^Li-MAS NMR spectra acquired for all NMC333 samples, for which two strong contributions are discernible. A rather narrow resonance was located at around 0 ppm. It can be assigned to diamagnetic impurities such as LiOH or Li_2_CO_3_. The main contribution was a broad group of resonances with large chemical shifts covering the range from 0 to 1000 ppm with a major broad band at approximately 550 ppm. The distribution of the signal intensity among the resonances was sample dependent. The Mg-doped sample showed the smallest 0 ppm peak, which increased in the undoped and the Al-doped sample. The highest 0 ppm intensity was also found for Fe- and Zn-doped NMCs. The broad contributions ranging from 0 to 1000 ppm were analyzed by means of a spectral deconvolution, which revealed the degree of cationic disorder (Table 2). Obviously, the shifts of the three resonances were approximately equidistant. In all samples, the intermediate resonance with a large chemical shift of about 550 ppm, which had the highest intensity, was assigned to the hyperfine interaction between Li^+^ nuclei and the unpaired electrons of Ni^2+^ and Mn^4+^ paramagnetic ions [57,58,59]. Note that the broadening of the peak at ~550 ppm suggests the less ordered local environment of Li on 3*b* sites.

Results shown in Figure 6a match well with those reported by Cahill et al. [58] who determined three resonances in the range from 200 to 800 ppm using ^6^Li NMR. These three NMR bands are assigned to Li^+^ ions in the interlayer slab of the NMC lattice with different TM distributions in the first cation coordination shell. The possible Ni^2+^/Co^3+^/Mn^4+^ arrangements exhibiting different chemical shifts are distributed by (a) 1:4:1 at low frequency (<300 ppm), (b) 2:2:2 at intermediate frequency (~550 ppm), and (c) 3:0:3 at high frequency (>700 ppm). Adopting these results, we interpreted the three major resonances identified in the deconvolution of our spectra to stem from lithium ions in the 3*b* site (Li layer). The observed hyperfine shifts can be readily explained by the three different local environments proposed by Cahill et al. [58]. Either one, two or three pairs of Ni^2+^ and Mn^4+^ ions are distributed among the six nearest neighbor positions. According to the nominal stoichiometry, the total number of ions of each type should be the same for all transition metals, leading to symmetric intensity distribution. This expectation is not in accordance with the results from the spectral deconvolution of the samples; only the Zn-doped NMC333 sample might be considered to show roughly a symmetric intensity distribution. Another NMR feature was a minor resonance occurring at around 1300 ppm attributed to Li^+^ ions in 3*a* site of the TM layers as a result of the cation mixing with Ni and Mn in first and second coordination shell. Thus, the Li/Ni exchange rate can be calculated by the ratio of the area of the smaller peak over the larger one including side bands (Table 2) and compared with data from Rietveld refinements. From spectra in Figure 6a, it is obvious that the Li/Ni exchange rate was lower for Al- and Fe-doped samples. There is a general agreement for the cation mixing diminution within the Al-doped LiNi_1/3_Mn_1/3_Co_1/3_O_2_ framework because Al prevents the presence of Li on the 3*a* site of TM layer and restrains Ni^2+^ in the Li plane [60]. Liu et al. [36] reported that the improved structural stability of NMC333 at low Al doping (*y* < 1/20), whereas Fe doping does not display such behavior even at low Fe content.

The Fe Mössbauer spectrum of LiNi_1/3_Mn_1/3_Co_1/3_O_2_ doped with Fe recorded at room temperature is shown in Figure 6b. It reveals a narrow doublet with an isomer shift of 0.327 ± 0.001 mm s^−1^ and a quadrupole splitting of 0.436 ± 0.002 mm s^−1^, which are characteristics of Fe^3+^ ions in high-spin state in octahedral oxygen coordination, as expected for the 3*a* site. No other contributions could be detected in the spectrum.

### 3.4. Electrochemical Properties

**Figure 7:**
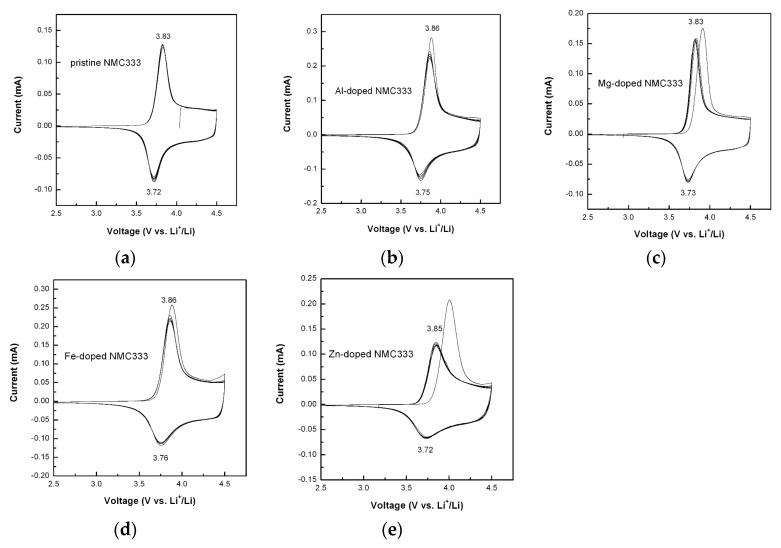
Cyclic voltammograms of parent (**a**) and doped LiNi_1/3_Mn_1/3_Co_1/3_O_2_ with Al (**b**), Mg (**c**), Fe (**d**) and Zn (**e**) at a sweep rate of 0.05 mV s^−1^ between 2.5 and 4.5 V versus Li^+^/Li^0^.

The effects of doping on the electrochemical properties were systematically investigated by cyclic voltammetry (CV) and galvanostatic charge-discharge (GCD) measurements in the voltage range of 2.5–4.5 V versus Li^+^/Li^0^. Figure 7 shows the CV profiles of parent and doped NMC333 oxides recorded at a sweep rate of 0.05 mV s^−^^1^. The pristine NMC333 electrode displayed a sharp anodic peak (delithiation) at 3.83 V and a cathodic peak (lithiation) at 3.72 V. Within the potential range 2.5–4.5 V, redox peaks are ascribed to the reaction of Ni^2+^ to Ni^3+/4+^, while Mn^4+^ is known to be electrochemically inactive and Co^3+/4+^ takes place at potentials above 4.6 V [19]. Except the pristine sample, all doped NMC333 samples exhibited the same features, i.e., a slight voltage shift of the anodic peak (Δ*E*_pa_) between the 1st and 2nd cycle, while the cathodic peak was almost unchanged. Δ*E*_pa_ appeared to be 20 mV for Al- and Fe-doped NMC; 90 and 160 mV for Mg- and Zn-doped electrodes, respectively. Similar behavior attributed to surface kinetics was reported by Riley et al. [61] for NMC333 coated with Al_2_O_3_ by atomic layer deposition. The forthcoming CV cycles show similar redox features, indicating a good reversibility for the lithiation/delithiation process, according to the relation [62]:

LiNi_1/3_Mn_1/3_Co_1/3_O_2_ ⇌ Li_1__−__x_Ni_1/3_Mn_1/3_Co_1/3_O_2_ + *x*Li^+^ + *x*e^−^ (2)

for which the redox couple Ni^2+/4+^ should be considered in the voltage range 2.5–4.5 V versus Li^+^/Li. Table 3 summarizes the redox peak potentials (*E*_pa_, *E*_pc_, Ni^2+/4+^) of the investigated NMC333 electrodes. The peak potential separation (PPS), i.e., expressed by *E*_p_ = *E*_pa_ − *E*_pc_, between anodic and cathodic potentials, corresponding to the Ni^2+/4+^ redox process, was measured in the range (100 ≤ *E*_p_ ≤ 130 mV), These results compare well with data in the literature [43,61]. Here, we can point out that the particle morphology also plays a crucial role. However, the PPS for the insertion type of materials also significantly depends on the scan rate in CV experiments. Lin et al. [43] reported high Δ*E*_p_ values (≈0.33 V at scan rate of 100 µV s^−1^) for Al-doped NMC synthesized via a sol-gel method. Similarly, Li et al. [63] mentioned Δ*E*_p_ values of about 0.3 V measured at scan rate of 0.1 mV s^−1^ for LiNi_1/3_Mn_1/3_Co_1/3−x_Al_x_O_2_ formed by large secondary particles (agglomerates ~1 µm). Wu et al. [37] reported a depressed *E*_p_ value (0.23 V at a scan rate of 0.1 mV s^−1^) for 1%-Al^3+^ substituting Ni^2+^ in NMC333 formed by nanoparticles (100–500 nm size) versus 0.42 V for the undoped sample.

**Figure 8:**
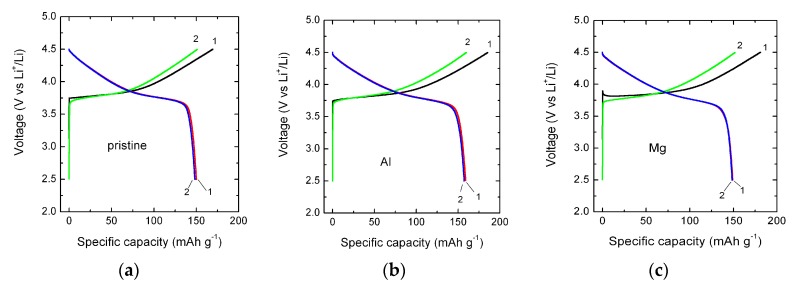

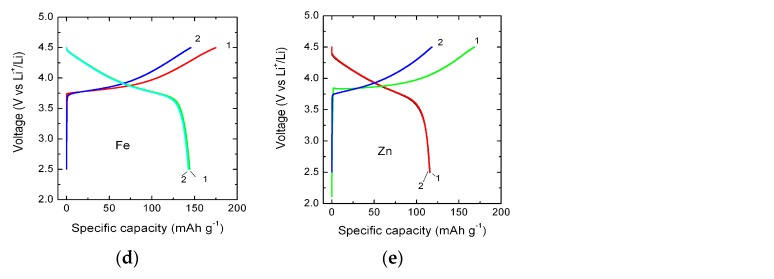
Charge-discharge profiles of pristine (**a**) and doped LiNi_1/3_Mn_1/3_Co_1/3_O_2_ with Al (**b**), Mg (**c**), Fe (**d**) and Zn (**e**) carried out at the 0.1C rate in the potential range 2.5–4.5 V versus Li^+^/Li^0^.

Figure 8 presents the galvanostatic charge–discharge curves of Li//NMC333 cells including parent and doped electrodes cycled at a constant current density of 0.1C at 25 °C. Analysis of these results showed that the Al-doped material exhibited better galvanostatic charge/discharge performance. At 0.1C, its initial specific discharge capacity was 160 mAh g^−1^ with a coulombic efficiency of ~85%. No significant change in the initial capacity value upon doping by Mg, similar to bare NMC333, of about 150 mAh g^−1^ was obtained for Mg-doped oxide. Both Fe and Zn doping negatively affect the initial capacities values. Fe-doped oxide delivered 146 mAh g^−1^, whereas Zn-doped material delivers a lower capacity of 118 mAh g^−1^. 

**Figure 9:**
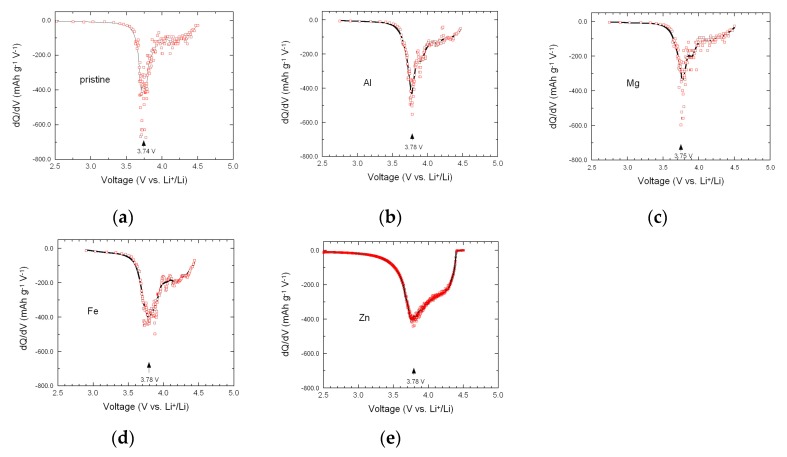
Differential capacity (−d*Q*/d*V*) plots of cycle #2 for pristine LiNi_1/3_Mn_1/3_Co_1/3_O_2_ (**a**) and *M*-doped LiNi_1/3_Mn_1/3_Co_1/3_O_2_ with Al (**b**), Mg (**c**), Fe (**d**) and Zn (**e**).

The incremental capacity (IC), i.e., differential capacity (−d*Q*/d*V*) versus V curve, can be considered as an electrochemical spectroscopy technique [17]. For instance, IC has been successfully applied to analyze the layered and spinel contribution in blended cathodes [64]. The IC curves were extracted from the galvanostatic discharge profiles (lithiation process) during the 2nd cycle to further characterize the electrochemical behavior of doped electrodes, as depicted in Figure 9. Each plot displays a main sharp peak at approximately 3.74–3.78 V versus Li^+^/Li^0^ and a broad voltage peak in the vicinity of 4.25 V, which are typical fingerprints of the Ni^2+/3+^ and Ni^3+/4+^ reactions. These results showed that both Al^3+^ and Fe^3+^ ions were electrochemically inactive for the cutoff charge-discharge voltages in the range 2.5–4.5 V.

**Figure 10:**
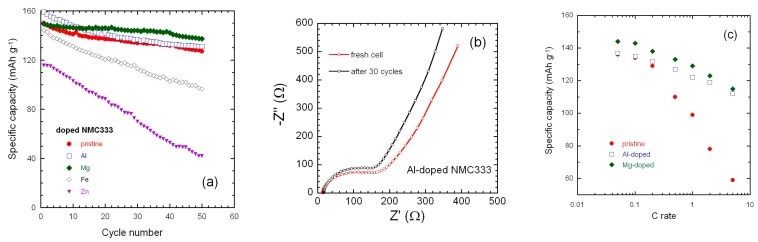
(**a**) Cyclability of parent and doped LiNi_1/3_Mn_1/3_Co_1/3_O_2_ cathode materials recorded at a 0.1C rate in the potential range 2.5–4.5 V versus Li^+^/Li^0^; (**b**) electrochemical impedance spectroscopy (EIS) results for a LiNi_1/3_Mn_1/3_Co_1/3_O_2_//Li cell before and after cycling at 0.1C rate; (**c**) rate capability of undoped and Al- and Mg-doped NMC333 electrodes after 30 cycles.

Figure 10a compares the cyclability of all samples cycled for 50 cycles at 0.1C. Both Al-and Mg-doped oxides show better rechargeability than bare NMC333, which delivers 85% of its initial capacity after 50 cycles, while Zn and Fe doping display worse results. Doped electrode materials with Mg, Al, Fe, and Zn retained specific capacity of 91%, 82%, 67%, and 36% of the initial values, respectively. Figure 10b presents the electrochemical impedance spectroscopy (EIS) measurements before and after cycling of a cell with Al-doped NMC333 as cathode material. Analysis of the Nyquist plots shows that at high frequency, the electrode/electrolyte resistance, *R*_s_ = 14.5 obtained by the intercept of the Z′ axis does not change upon cycling. In contrast, the charge transfer resistance (*R*_ct_) corresponding to the electrochemical reaction at solid/electrolyte interface (represented by the depressed semicircle at medium frequency region) decreased from 192 for the fresh cell to 165 after 30 cycles. This decrease is attributed to the cell formation occurring after few cycles of charge–discharge. Rate capability was tested in the range from 0.05 C to 5C for the 30th cycle. Results shown in Figure 10c also demonstrated the beneficial effect of Al and Mg doping after 30 cycles of charge-discharge. The specific discharge capacity of pristine NMC333 decreased dramatically down to 59 mAh g^−1^ with increasing current density to 5C, while both capacities of Al- and Mg-doped NMC333 maintained at approximately 112 mAh g^−1^.

## 4. Discussion

In this work, we chose two dopants, Al and Fe, which substituted for Co. Reducing the number of cobalt ions did not penalize per se the capacity when the cell is operating in the voltage range 2.5–4.5 V, because the redox Co^3+/4+^ reaction occurred at a potential >4.6 V. The effects on the electrochemical properties are, however, totally different. The large strain field <e^2^> opposed the diffusion of lithium, with the consequence that some of the Li are immobilized so that the initial capacity was smaller than in the pristine sample, and the kinetics were slower, with the consequence that the rate capability was degraded. Moreover, the large strain field gives evidence of a poorer structural stability, implying a rapid decrease of the discharge capacity as a function of the cycle number. To the contrary, Al doping improved significantly the electrochemical properties. The two other dopants, Mg and Zn, substituted mainly on the Ni site and created also anti-site defects, so that a fraction of them also occupy the Li-sites. However, these dopants have also very different impact on the electrochemical properties. Zn doping degrades the electrochemical properties for the same reasons invoked for Fe doping: it provokes a large strain field that immobilizes some of the lithium ions, slows the kinetics, and decreases the structural stability. On another hand, Mg doping improves the electrochemical properties, but differently. The lithium ions in the anti-site defects do not participate in the electrochemical process [17]. Therefore, the more anti-site defects that exist, the smaller the capacity of the battery. The overall concentration of anti-sites is in the order: pristine > Mg-doped > Al-doped, after the results in Table 1. We then expect the initial capacity at a low rate in the opposite order: Pristine < Mg-doped < Al-doped, in agreement with the initial capacity at 0.1C in Figure 10a. The reason is that, even though Mg- oping is efficient to suppress the Li–Ni anti-sites, the Mg–Ni anti-site is inevitable, because Mg^2+^ and Li^+^ have almost the same ionic radius. As a consequence, the overall anti-site concentration in the Mg-doped sample was larger compared to Al-doping, despite the absence of Li–Al anti-sites. On another hand, the significant strain field associated with Al-doping evidenced in Figure 2 and Table 1 was responsible for reduced cycle ability, in agreement with Figure 10a, where the two curves of the capacity versus number of cycles cross each other, so that the capacity was larger in the Mg-doped sample after 12 cycles. The beneficial effect of Mg doping is in good agreement with the result of Luo et al. [65], reporting the beneficial effect of Mg substitution on the degree of cation mixing, although we do not agree with the assumption that Mg substituted for Mn, as claimed in this prior work, as the apparent increase in transport properties, can be attributed to the modification of the microstructure and the slight decrease of the NMC333 particle sizes with Al- and Mg-doping concentrations (> 0.02) [66]. The change in the microstructure was related to the increase of the interlayer distance *I(LiO_2_)* from 2.6393 Å in pristine NMC to 2.6508 and 2.6817 Å in Al- and Mg-doped NMC, respectively (see Table 1). The increased *I(LiO_2_)* results in better mobility of Li^+^ ions in the NMC framework to enhance the rate capability. In contrast, different researchers have shown contradictory results. For example, in Reference [29], the authors reported that Mg dopant in Li(Ni_1/3_Co_1/3_Mn_1/3_)O_2_ cathodes synthesized by hydroxide coprecipitation method did not exhibit improvement and stated an increase of undesirable reactions between the electrode and the electrolyte inducing larger capacity fade. Hence, this lack of improvement seems to be due to the morphology of the NMC powders having very large particle size distribution. Several authors [67,68] proposed that the enhanced cycling stability of Mg substitution samples was attributed to the Mg ions incorporation into interlayer planes due to the similar ionic radii of Li^+^ and Mg^2+^. This is in opposition with the present Rietveld refinements, which determine a small concentration of 1.3% for Mg^2+^ on Li sites and a weak cationic mixing rate of 0.24%. 

The improvement of Al-doped electrode relative to bare NMC333 cathode materials is commonly attributed to an enlarged Li layer spacing and a reduced degree of cation mixing [36,37,38,39]. Presently, the as-prepared Li(Ni_1/3_Mn_1/3_Co_1/3_)_0.94_Al_0.06_O_2_ powders show limited defect in the Li plane, 2.4% Ni^2+^ and 1.3% Al^3+^ on Li site. Note that, in this work, for a relevant comparison, special attention was taken to prepare powders having the same morphology, particle size, and size distribution by adjusting the temperature and duration of the annealing process [69]. Both the charge and discharge voltage plateaus of Al-doped NMC333 were higher than those of the pristine electrode. First-principles calculations also predicted the potential increase with the substitution of Co with Al [70]. This phenomenon, experimentally observed by Julien et al. [71] in Al-doped LiNi_0.5_Co_0.5_O_2_ and by Liu et al. [36] in LiNi_1/3_Co_1/3_Mn_1/3_O_2_, was due to the drop in the chemical potential of the material. The lowering delithiation potential (end of charge) for Fe-doped LiNi_1/3_Co_1/3_Mn_1/3_O_2_ was also identified by Meng et al. [40], while the raise in the delithiation voltage for Al^3+^ doping of NMC materials was reported by Liu et al. [36]. Our results are in good agreement with those reported by Samarasingha et al [72] on Li(Ni_1/3_Mn_1/3_Co_(1/3−x)_Fe_x_)O_2_ with *x* = 0.11 for which an initial specific discharge capacity of ca. 126 mAh g^−1^ was measured at 36 mA g^−1^ current density. In conclusion, the substituted elements slightly enlarged the interlayer spacing favoring a high degree of ordering and improving the ionic transport kinetics, i.e., Li^+^ ions are faster in doped materials than in the undoped lattice. Less Li^+^/Ni^2+^ cation mixing that favors kinetics was also reported for doped NMC materials [26,73]. The poor electrochemical performance of NMC333 is in agreement with a previous report [74], and is due to the important local distortions, which oppose the Li motion and decrease the lattice stability.

## 5. Conclusions

A simple sol-gel method assisted by EDTA as chelator was carried out to prepare LiNi_1/3_Mn_1/3_Co_1/3_O_2_ cathode materials doped with Al, Mg, Fe, and Zn. The regular morphology and almost identical particle sizes allow for an accurate comparison of electrochemical performances. Considering the intensity ratios of XRD reflections and performing Rietveld refinements, the structural properties showed that not only the degree of cation mixing but also the strain field were sensitive to the doping element. Experimental results revealed that both Mg- and Al-doped NMC333 electrodes delivered the best long-term cyclability and rate capability due to the minimum occupancy of foreign ions in the Li plane. However, Mg doping was the best, because it minimized the local distortions in the lattice, so that the cycle ability was better, even though the discharge capacity in the first cycles was larger with Al-doping.

## Figures and Tables

**Table 1:** Results of Rietveld refinements for undoped Li(Ni_1/3_Mn_1/3_Co_1/3_)O_2_ and doped Li(Ni_1/3_Mn_1/3_Co_1/3_)_1−x_*M*_x_O_2_ (*x* = 0.06) (*M* = Fe, Al, Mg, Zn).

Crystal Data	Parent	Doping Element (*M*)			
		Mg	Al	Fe	Zn
Lattice parameters	-	-	-	-	-
*a* (Å)	2.856 (2)	2.865 (4)	2.858 (5)	2.867 (1)	2.864 (7)
*c* (Å)	14.249 (5)	14.259 (7)	14.255 (9)	14.276 (7)	14.243 (1)
*c/a*	4.980 (1)	4.976 (3)	4.987 (1)	4.979 (4)	4.964 (9)
*V* (Å^3^)	100.65	101.39	100.88	101.62	101.22
*L*_c_ (nm)	18.7	17.3	25.6	14.8	15.8
<e^2^> × 10^−5^ (rd^2^)	0.2899	0.4221	0.1303	0.8743	0.8458
*I_(_*_003)_/*I*_(104)_	1.61	1.83	1.53	1.49	1.22
(*I*_(006)_ + *I*_(102)_)/*I*_(101)_	0.47	0.42	0.49	0.58	0.56
Residuals	-	-	-	-	-
*R_p_(%)*	8.17	8.44	8.92	9.15	10.21
*R_wp_(%)*	9.01	9.69	11.07	11.50	13.81
*R_F_*	1.68	2.16	3.16	5.31	4.12
Occupancy (Occ)	-	-	-	-	-
Ni^2+^ on Li-site	0.0241	0.0024	0.0142	0.0129	0.0113
*M* on Li-site	-	0.0127	-	-	0.0222
(0,0,z) for *O_2_*	0.25921	0.2607	0.25964	0.2585	0.25230
*S(MO_2_)*^a^ (Å)	2.1139	2.0714	2.1011	2.1367	2.2882
*I(LiO_2_)*^b^ (Å)	2.6393	2.6817	2.6508	2.6222	2.4595

^a^*S(MO_2_) = 2((1/3)* − *Z_oxy_)c* is the thickness of the metal–O_2_ planes; ^b^ *I(LiO_2_) = c/3 − S(MO_2_)* is the thickness of the inter-slab space.

**Table 2:** Deconvolution of sample spectra. Calculated areas include all significant sideband contributions.

Dopant	Shift (ppm)	*I*_rel_	Shift (ppm)	*I*_rel_	Shift (ppm)	*I*_rel_	%Li/Ni Exchange
None	274.9	21.1	541.4	66.9	747.3	12.0	3.11
Al	277.1	26.2	539.8	62.0	731.0	11.7	1.65
Mg	289.0	28.5	533.4	52.1	716.6	19.4	3.03
Fe	283.5	7.0	563.4	54.9	776.1	38.1	2.08
Zn	217.9	28.8	541.0	49.7	743.0	21.5	2.74

**Table 3:** The oxidation (*E*_pa_) and reduction (*E*_pc_) peak potential and the corresponding difference Δ*E*_p_ obtained from CV data (scan rate of 0.05 mV s^−1^) of doped NMC333 cathodes for the 2nd cycle.

Potential (V Versus Li^+^/Li)	Doping Element				
	Pristine	Al	Mg	Fe	Zn
*E*_pa_	3.83	3.86	3.83	3.86	3.85
*E*_pc_	3.72	3.75	3.73	3.76	3.72
Δ*E*_p_	0.11	0.11	0.10	0.10	0.13
